# Potential of autosomal crossover count to enhance additive genetic variance in pig breeding programs

**DOI:** 10.1186/s12711-026-01114-0

**Published:** 2026-11-10

**Authors:** Olivia Katz, Yvonne C. J. Wientjes, Anne C. M. Jansen, Mario P. L. Calus

**Affiliations:** Animal Breeding and Genomics, Wageningen University and Research, Wageningen, 6700 AH The Netherlands

## Abstract

**Background:**

Crossovers can break the linkage between loci, thereby creating novel allele combinations and releasing genetic variation. However, empirical studies evaluating their potential use in livestock breeding programs remain scarce. In this study, we investigated the link between autosomal crossover count (ACC) and additive genetic variance for reproduction and production traits in pigs. Using univariate and bivariate sire models, individuals were grouped into five bins according to the number of paternal autosomal crossovers they inherited, as estimated in a previous study. Within each bin, sire additive genetic variance was estimated. The analysis included more than 50,000 records for total number born, number born alive, and number of stillborn piglets; over 14,000 records for number of teats; and more than 35,000 records for standardized average daily gain.

**Results:**

For all traits, the 95% confidence interval of the overall estimated sire additive genetic variance overlapped with the estimates obtained for each ACC bin. Likewise, the 95% confidence interval for differences in $\:{\widehat{\sigma\:}}_{a,sire}^{2}$ between consecutive bins also overlapped in almost all cases. These results indicate that the observed number of crossovers did not significantly affect the $\:{\widehat{\sigma\:}}_{a,sire}^{2}$. Nevertheless, trait-specific patterns were apparent. For three traits, the $\:{\widehat{\sigma\:}}_{a,sire}^{2}$ was highest at intermediate ACC ranges, whereas for the remaining traits it reached its maximum at either low or high ACC ranges. Despite the lack of statistically significant differences, the bins with the lowest and highest $\:{\widehat{\sigma\:}}_{a,sire}^{2}$ differed by an average of 57% across traits.

**Conclusions:**

In conclusion, ACC may be of potential interest in breeding programs as a trait worth further investigation, although the present results do not provide significant evidence to support its direct use for the analyzed ACC ranges.

**Supplementary Information:**

The online version contains supplementary material available at https://doi.org/10.1186/s12711-026-01114-0.

## Background

Meiotic recombination enables the exchange of genetic information between homologous chromosomes through events known as crossovers [1, 2]. Therefore, crossovers can break up the link between loci, and produce new combinations of alleles, which potentially may increase genetic variation within populations [2, 3]. Genetic variation is needed for the adaptation of a population to a changing environment, the maintenance of genetic diversity [2, 4] and it is also advantageous for animal breeding purposes [5, 6]. Because crossover events between the two parents are independent, paternal and maternal autosomal crossovers transmitted to an individual are also independent. In our study, autosomal crossovers refer to the crossovers that a parent transmits to an offspring during the formation of gametes, following [7], and also to those crossovers occurring in all chromosomes except for the sex chromosomes. Hence, because parents in animal breeding are typically associated with multiple offspring, a single parent often has multiple ACC observations.

Previous studies reported substantial variation in the number of autosomal crossovers among individuals in species like pigs, cattle, sheep, Atlantic Salmon, mouse and human [1, 8–11]. In pigs, this variation has been shown to be partially heritable [7, 12]. In particular, heritability estimates of ACC in pigs were reported to range between 0.07 and 0.11 in sows and between 0.04 and 0.05 in boars [7, 13, 14]. These results indicate that ACC might be a trait to target in breeding programs to increase genetic gain for other breeding goal traits by creating novel combinations of alleles by breaking the link between nearby loci. However, despite of these findings, studies [5, 7, 14] reported that some precautions might be needed in terms of added benefits of incorporating ACC into breeding programs.

We therefore hypothesise that individuals transmitting higher ACC might increase the additive genetic variance of breeding goal traits by breaking up the link between loci. A simulation study reported that in order to achieve significant increases in genetic gains in other breeding goal traits, the number of autosomal crossovers should be increased by 10 to 20 times, which might lead to biological constrains associated to the limitations of increasing the number of crossovers at that scale [5]. However, the extent to which autosomal crossovers may impact the release of genetic variance using empirical data remains largely unexplored. While variation in ACC among pigs is known to be partially heritable [7], and a trait’s additive genetic variance is central to obtain genetic gain [3, 5], it is still unclear whether and how differences in ACC across the population may affect the additive genetic variance for breeding traits [15].

Hence, this study aimed to investigate the link between ACC and the additive genetic variance using empirical data for various reproductive and production traits in pigs. In order to study this, we primarily estimated sire additive genetic variance ($\:{\widehat{\sigma\:}}_{a,sire}^{2}$) for the breeding goal traits using offspring’s phenotypic records, grouping observations by the number of paternal autosomal crossovers inherited by each daughter. Complementary analyses were also performed using dam models with maternal crossovers. For the analysis we used three different models and applied them separately in a sow line, primarily selected for fertility traits, and in a boar line, primarily selected for production traits. We used the observations for those individuals with records for ACC and for the breeding goal traits. This included over 50,000 observations for the traits total number of piglets born, number of piglets born alive and number of stillborn piglets; over 14,000 observations for number of teats and over 35,000 observations for the trait standardised average daily gain.

## Materials and methods

In this section, we first describe the datasets used for the analyses. This is followed by the method used to determine ACC, a description of the used phenotypes of breeding goal traits, and the statistical models applied.

### Datasets and ACC in parent-offspring pairs

Data were provided by Topigs Norsvin and comprised genomic data of two commercial pig lines: a Large White sow line, containing 246,630 individuals, and a synthetic boar line, containing 128,667 individuals. For all individuals, 23,588 Single Nucleotide Polymorphisms (SNPs) genotype data from an Illumina Geneseek custom 25K SNP chip was available, with SNP positions mapped to the Sscrofa11.1 reference genome. All SNPs on the sex chromosome were excluded for our analysis and an initial quality control removed individuals and SNPs with more than 1% missing genotypes. To determine the number of crossovers, we only retained genotyped families in which an individual has at least five offspring and both parents and four grandparents genotyped to ensure sufficient information for haplotype phasing. After filtering, the final datasets included 8265 families with a pedigree of 78,446 individuals in the sow line, and 5642 families and a pedigree of 47,549 individuals in the boar line, genotyped for 20,713 SNPs (Table 1).

ACC for all individuals were previously determined in three steps using LINKPHASE3 [16], as described in [17]. The first step involved phasing the genotypes to determine the parental origin of haplotypes. During the running of LINKPHASE3 [16], parent-offspring pairs with more than 50 opposing homozygotes or Mendelian errors were removed. In total, there were 334 cases in the sow line and 96 cases in the boar line, and the respective parent or parents were set to missing in the pedigree. This step resulted in 94 (sow line) and 49 (boar line) unique sires and 120 (sow line) and 55 (boar line) unique dams of individuals that were removed from the pedigree. Also, we excluded SNPs with more than 5 Mendelian errors. In total, we excluded 290 SNPs in the sow line and 169 SNPs in the boar line, leaving 20,423 and 20,544 SNPs, respectively. As a second step, LINKPHASE3 [16] was run using 1 iteration to corroborate that each parent-offspring pair had under 50 Mendelian errors. Then, we analysed possible map errors (i.e. incorrect SNP positions) with the output file Map Confidence Score (MCS) provided by the software. The MCS indicates how reliable each SNP’s position is on the linkage map [16]. This step implied the manual exclusion of 7 SNPs in the sow line and 2 SNPs in the boar line. As a result, there was a total of 20,415 SNPs in the sow line, and 20,541 SNPs in the boar line. As a third step, we re-run LINKPHASE3 [16] using the cleaned pedigree and SNP dataset to estimate the crossover count by chromosome for all parent-offspring pairs of both pig lines. Following haplotype phasing, LINKPHASE3 identifies crossover events by examining inheritance patterns between parent–offspring pairs. The number of crossovers per chromosome for each pair is obtained directly from the output and used to estimate the ACC. A detailed description of the ACC determination procedure is provided in the flowchart in Additional File 1, Figure S2 [17]. The ACC was defined as the number of autosomal crossover events that a parent transmitted to an offspring. The total number of parent-offspring pairs with ACC estimates were 150,228 for the sow line and 91,371 for the boar line, and the total number of animals with identified crossovers per sex and line are in Table 1.

**Table 1:** Description of the two datasets used for ACC identification after quality control

Line	Sow line	Boar line
Number of genotyped families	8265	5642
Number of dam-offspring pairs with ACC estimation	74,223	45,182
Number of sire-offspring pairs with ACC estimation	76,005	46,189
Number of total parent-offspring pairs with ACC estimation	150,228	91,371
Number of male offspring	33,838	25,733
Number of female offspring	42,832	19,979
Number of offspring with unknown sex	1776	1837
Number of unique sires	840	588
Number of unique dams	7776	4891

As an extra filter, and in order to increase the overall accuracy of the phased individuals [4], we now only used ACC estimates from individuals that had a least one genotyped maternal grandparent and one genotyped paternal grandparent. Although LINKPHASE3 was run on pedigrees comprising the filtered genotyped families, crossover estimates were generated for all parent-offspring pairs that are informative for phasing within those families. This includes parent-offspring pairs involving individuals such as sires and dams, whose own grandparental pedigree is incomplete. As a result, we used a final dataset of 144,912 estimated ACC records in the sow line and 87,984 in the boar line.

As ACC represents the number of autosomal crossovers transmitted from a parent to an offspring, each record corresponds to a single transmission event for a parent-offspring pair, and parents can contribute multiple observations across offspring. Although each ACC record is linked to a specific parent-offspring pair, ACC reflects crossover events occurring during meiosis in the transmitting parent. This results in a repeated-measures structure, which is appropriately modelled using a sire (or dam) model to capture variation in autosomal crossovers transmitted by the sire (or dam). Note that we did not use the total number of crossovers received by an individual, obtained by combining its paternal and maternal crossover counts, because this would result in a single recombination value for each offspring and would not allow us to estimate variation in additive genetic variance across the range of crossover counts. For that reason and because sires usually are linked to more offspring than dams and thus have more observations, we mostly focus on ACC that came from the sire (from now on referred to as ACCs) in our study. The sow line included 72,456 sire-offspring pairs with ACCs observations, while the boar line included 43,992 of such pairs. As mentioned before, although the main analyses were based on sire models, we also conducted dam-model analyses to compare paternal and maternal ACC patterns.

### Phenotypes

Phenotypic records for five breeding goal traits were used to study the dependency of their $\:{\widehat{\sigma\:}}_{a,sire}^{2}$ on ACCs. From these breeding goal traits, three were fertility traits (only recorded in the sow line), one was a trait with high heritability (only recorded in the sow line), and one was a production trait (recorded in both the sow and boar line). Given the expected dependency of fertility on ACC, we focused primarily on fertility traits to study their dependency on ACCs, while including one production trait for comparison. The included fertility traits were total number of piglets born (TNB), number of piglets born alive (NBA) and number of stillborn piglets (NSB). The trait with high heritability was number of teats (NT), which is defined as the total number of teats on both sides of the body and is recorded at 1 day of age; and the production trait was standardised lifetime daily gain (SLDG), defined as the average weight gain from birth until the end of the testing period (grams/day). The testing period refers to the post-lactation growth phase, ending at approximately 180–200 days of age and 100–110 kg. To correct for that variation, the measurements were standardised. In farms with a nursery phase, the testing period includes both the nursery and finishing phase; in farms without nursery, it corresponds to the finishing phase only. The datasets and phenotype cleaning process are described in the following sections in more detail and was performed using R statistical software (v4.4.1; R Core Team 2024) [18].

#### Fertility traits

For TNB, NBA, and NSB, there was a total initial number of 437,865 observations from 128,441 unique individuals. For the quality control, we started with verifying that TNB = NBA + NSB, which was indeed the case for all observations. After that, we removed 22 outliers for TNB and 13 for NBA, using the criteria that the phenotype differed > 4 SD from the mean. This resulted in a final dataset of 437,843 observations for TNB (128,439 individuals) with a mean of 16.09 (SD = 3.97) and of 437,852 for NBA (128,436 individuals) with a mean of 14.71 (SD = 3.79).

For NSB, we didn’t consider removing outliers based on 4 SD, because the trait was not normally distributed and the distribution of the trait had a right skewed distribution. For that reason, values above the 99.9th percentile (NSB > 15; 368 observations) were removed, leaving 437,497 observations from 128,393 individuals (mean = 1.36, SD = 1.83). Additionally, for NSB, phenotypes were transformed using a Box-Cox transformation (from now on, NSBbc), after adding 1 to all records to avoid 0 values, to improve the approximation to normality of the residuals [19].

#### Number of teats

For NT, the initial dataset contained 148,390 observations (one per animal). For this trait, we first removed 52,208 observations with NT = 0, resulting in 96,182 sows with observations. Then, 51 outliers (> 4 SD) were removed, leaving a total of 96,131 observations (mean = 16.11, SD = 1.07).

#### Production traits

For SLDG, each animal had one observation, and the initial dataset contained 211,355 observations for the sow line. A total number of 53 outliers (> 4 SD) were removed, resulting in a dataset with 211,302 observations (mean = 685.62, SD = 67.50). The boar line contained 129,833 observations, from which a total number of 104 outliers (> 4 SD) were removed, resulting in a dataset with 129,729 observations (mean = 822.56, SD = 66.14).

### Phenotypic datasets retained for the analysis

For the analysis of all traits, we retained only sire-offspring pairs for which the sire ACCs and the corresponding offspring phenotype were available. In the sow line, for the traits TNB and NBA, the final dataset contained 50,860 observations from 15,164 sows; for NSB, there were 50,824 observations from 15,158 sows; for NT, the dataset included 14,927 sows; and for SLDG, the datasets contained 39,757 individuals between boars and sows. For SLDG in the sow line, we excluded four additional observations because they belonged to fixed-effect levels with only one observation per level. This resulted in a final dataset of 39,753 observations for SLDG in the sow line. For the boar line, the SLDG dataset contained 35,168 individuals.

### Modelling sire additive genetic variance

We aimed to study the potential dependency of $\:{\widehat{\sigma\:}}_{a,sire}^{2}$, estimated from offspring’s phenotypes, on ACCs, for each trait. To investigate this, we started by fitting univariate sire models, for which we divided the offspring’s phenotypic data into five ACCs-defined bins and estimated $\:{\sigma\:}_{a,sire}^{2}$ within each bin. Bins were defined to contain a similar number of observations, with non-overlapping ACCs ranges between bins. Description of the bins for each trait are in Table 2. Thereafter, we fitted a multivariate analysis using all five ACCs-defined bins as separate traits. Unfortunately, this multivariate model did not converge, likely due to its complexity and overparameterization and because of difficulties in maintaining positive definiteness of the sire covariance matrix. Hence, we decided to run all 10 combinations of the five bins in bivariate models. In addition, because crossovers occur independently between progenitors and ACC differ between sires and dams, we also performed univariate analysis using the crossovers transmitted by the dams of the individuals, that were also obtained in our previous study [17] (description of the bins created for univariate dam models are in [Additional file 1, Table S3]). This analysis was used to compare the patterns between $\:{\widehat{\sigma\:}}_{a,sire}^{2}$ and $\:{\widehat{\sigma\:}}_{a,dam}^{2}$, using the same fixed and random effects as in the sire models described above. All models were fitted using ASReml 4.2 [20]. Each of the models is hereafter explained in more detail.

**Table 2:** Description of the bins created for the univariate and bivariate sire models

Trait	Bin 1		Bin 2		Bin 3		Bin 4		Bin 5	
	*N*	Min-Max ACCs	*N*	Min-Max ACCs	*N*	Min-Max ACCs	*N*	Min-Max ACCs	*N*	Min-Max ACCs
TNB/NBA	7174	7–14	9228	15–16	11,597	17–18	10,744	19–20	12,117	21–33
NSBbc	7166	6–14	9219	15–16	11,592	17–18	10,739	19–20	12,108	21–33
NT	2091	5–14	2772	15–16	3538	17–18	3054	19–20	3472	21–33
SLDG (sow line)	5487	5–14	7200	15–16	9243	17–18	8399	19–20	9429	21–34
SLDG (boar line)	6475	5–14	7479	15–16	8283	17–18	6795	19–20	6136	21–33

### Univariate models

For each trait, we firstly estimated $\:{\sigma\:}_{a,sire}^{2}$ using all the observations without accounting for bins. Then, we estimated $\:{\sigma\:}_{a,sire}^{2}$ separately for all observations in each of the five bins of ACC. We compared $\:{\widehat{\sigma\:}}_{a,sire}^{2}$ per bin with those obtained from the univariate analyses using all the observations. The levels of the different fixed and random effects are shown in Table 3. Only fixed effects that were statistically significant or biologically relevant, such as ACCs, were retained in the final model. For each univariate analysis, the sire model was specified as follows:

- For NBA, TNB and NSBbc:

1 $$\:\mathbf{y}=\mathbf{X}\mathbf{b}+{\mathbf{Z}}_{\mathbf{h}\mathbf{y}\mathbf{s}}{\mathbf{c}}_{\mathbf{h}\mathbf{y}\mathbf{s}}+{\mathbf{Z}}_{\mathbf{p}\mathbf{e}}\mathbf{p}\mathbf{e}+{\mathbf{Z}}_{\mathbf{s}}\mathbf{s}+\mathbf{e},$$

$$\:{\mathbf{c}}_{\mathbf{h}\mathbf{y}\mathbf{s}}\:\sim\:N(0,\:\mathbf{I}{\sigma\:}_{hys}^{2}),$$

$$\:\mathbf{p}\mathbf{e}\:\sim\:N(0,\:\mathbf{I}{\sigma\:}_{pe}^{2}),$$

$$\:\mathbf{s}\:\sim\:N(0,\:\mathbf{A}{\sigma\:}_{a,sire}^{2}),$$

$$\:\mathbf{e}\:\sim\:N(0,\:\mathbf{I}{\sigma\:}_{e}^{2}),$$

Where $\:\mathbf{y}$ was the vector of phenotypic observations, $\:\mathbf{X}$ was the incidence matrix relating observations to fixed effects, $\:\mathbf{b}$ is the vector of fixed effects, $\:{\mathbf{Z}}_{\mathbf{h}\mathbf{y}\mathbf{s}}$ was the incidence matrix relating observations to the herd-year-season at farrowing, $\:{\mathbf{c}}_{\mathbf{h}\mathbf{y}\mathbf{s}}$ was the vector the herd-year-season at farrowing effect; $\:{\mathbf{Z}}_{\mathbf{p}\mathbf{e}}$ was the incidence matrix relating observations to the permanent environmental effects, $\:\mathbf{p}\mathbf{e}$ was the vector of permanent environmental effect; $\:{\mathbf{Z}}_{\mathbf{s}}$ was the incidence matrix relating observations to the random sire genetic effects, $\:\mathbf{s}$ is the vector of sire random effects -or ½ the breeding values of the sires-, $\:\mathbf{e}$ is the vector of residuals. $\:\mathbf{A}$ is the pedigree-based relationship matrix among sires, $\:\mathbf{I}$ is an identity matrix describing the variance between residuals ($\:{\sigma\:}_{e}^{2})$.

- For NT:

2 $$\:\mathbf{y}\:=\mathbf{X}\mathbf{b}+{\mathbf{Z}}_{\mathbf{h}\mathbf{y}\mathbf{s}\mathbf{b}\mathbf{i}\mathbf{r}\mathbf{t}\mathbf{h}}{\mathbf{c}}_{\mathbf{h}\mathbf{y}\mathbf{s}\mathbf{b}\mathbf{i}\mathbf{r}\mathbf{t}\mathbf{h}}+{\mathbf{Z}}_{\mathbf{d}\mathbf{e}}\mathbf{d}\mathbf{e}+{\mathbf{Z}}_{\mathbf{s}}\mathbf{s}+\mathbf{e},$$

$$\:{\mathbf{c}}_{\mathbf{h}\mathbf{y}\mathbf{s}\mathbf{b}\mathbf{i}\mathbf{r}\mathbf{t}\mathbf{h}}\:\sim\:N(0,\:\mathbf{I}{\sigma\:}_{hysbirth}^{2}),$$

$$\:\mathbf{d}\mathbf{e}\:\sim\:N(0,\:\mathbf{I}{\sigma\:}_{de}^{2}),$$

$$\:\mathbf{s}\:\sim\:N(0,\:\mathbf{A}{\sigma\:}_{a,sire}^{2}),$$

$$\:\mathbf{e}\:\sim\:N(0,\:\mathbf{I}{\sigma\:}_{e}^{2}),$$

Where $\:{\mathbf{Z}}_{\mathbf{h}\mathbf{y}\mathbf{s}\mathbf{b}\mathbf{i}\mathbf{r}\mathbf{t}\mathbf{h}}$ was the incidence matrix relating observations to the herd-year-season at birth, $\:{\mathbf{c}}_{\mathbf{h}\mathbf{y}\mathbf{s}\mathbf{b}\mathbf{i}\mathbf{r}\mathbf{t}\mathbf{h}}$ was the vector of the herd-year-season at birth effect, and $\:\mathbf{d}\mathbf{e}$ was the vector of non-genetic dam effects.

For SLDG:

3 $$\:\mathbf{y}=\mathbf{X}\mathbf{b}+{\mathbf{Z}}_{\mathbf{p}\mathbf{g}}\mathbf{p}\mathbf{g}+{\mathbf{Z}}_{\mathbf{l}\mathbf{i}\mathbf{t}\mathbf{t}\mathbf{e}\mathbf{r}}\mathbf{l}\mathbf{i}\mathbf{t}\mathbf{t}\mathbf{e}\mathbf{r}+{\mathbf{Z}}_{\mathbf{s}}\mathbf{s}+\mathbf{e},$$

$$\:\mathbf{p}\mathbf{g}\:\sim\:N(0,\:\mathbf{I}{\sigma\:}_{pg}^{2}),$$

$$\:\mathbf{l}\mathbf{i}\mathbf{t}\mathbf{t}\mathbf{e}\mathbf{r}\:\sim\:N(0,\:\mathbf{I}{\sigma\:}_{litter}^{2}),$$

Where $\:{\mathbf{Z}}_{\mathbf{p}\mathbf{g}}$ was the incidence matrix relating observations to the pen group effect, $\:\mathbf{p}\mathbf{g}\:$was the vector of the pen group effect; $\:{\mathbf{Z}}_{\mathbf{l}\mathbf{i}\mathbf{t}\mathbf{t}\mathbf{e}\mathbf{r}}$ was the incidence matrix relating observations to the litter effect, $\:\mathbf{l}\mathbf{i}\mathbf{t}\mathbf{t}\mathbf{e}\mathbf{r}$ was the vector of the litter effect.

For NT and SLDG, the distributional assumptions of the sire genetic effects and the residuals were analogous to those in the previous traits.

**Table 3:** Description of the datasets and fixed and random effects for each trait ^a)^

Effect	Trait					
	NBA	TNB	NSBbc	NT	SLDG (sow line)	SLDG (boar line)
Number of individuals	15,164	15,164	15,158	14,927	39,753	35,168
ACCs	28	27	29	27	29	30
ID of the sires	538	538	538	418	547	407
ID of the dams for permanent environmental effect/non genetic dam effects	4323	4323	4323	4001		
Parity number	8	8	8			
HYS^a)^ at farrowing	3664	3664	3664			
Type of litter being gestated	3	3	3			
HYS^a)^ at birth				455		
Sex^b)^				1	2	2
Line^b)^					1	1
HYS^a)^ at the end of testing					819	316
Pen group					7277	7927
Birth weight class					19	1
Farm at birth					41	4
Farm at the end of testing					43	6
Litter ID at birth					7438	5588

^a^ Herd-year-season; ^b)^For SLDG in both lines, two interactions were fitted between line and Sex, and between Farm at birth and at the end of the testing period

### Bivariate models

We also used bivariate models to study the dependency on the $\:{\widehat{\sigma\:}}_{a,sire}^{2}$ of the breeding goal traits with ACCs, using the same bins as in the univariate models. We combined the five bins in ten different bivariate analyses. Hence, bivariate models allowed to model two different ACCs classes as two different correlated traits. For each bivariate analysis, the model was specified as follows:

- For NBA, TNB and NSBbc:

4 $$\eqalign{ \left[ {\matrix{ {{{\bf{y}}_1}} \cr {{{\bf{y}}_2}} \cr } } \right] & = \left[ {\matrix{ {{{\bf{X}}_1}} & 0 \cr 0 & {{{\bf{X}}_2}} \cr } } \right]\left[ {\matrix{ {{{\bf{b}}_1}} \cr {{{\bf{b}}_2}} \cr } } \right] + \left[ {\matrix{ {{{\bf{Z}}_{{\bf{hys}},1}}} & 0 \cr 0 & {{{\bf{Z}}_{{\bf{hys}},2}}} \cr } } \right]\left[ {\matrix{ {{{\bf{c}}_{{\bf{hys}},1}}} \cr {{{\bf{c}}_{{\bf{hys}},2}}} \cr } } \right] \cr & + \left[ {\matrix{ {{{\bf{Z}}_{{\bf{pe}},1}}} & 0 \cr 0 & {{{\bf{Z}}_{{\bf{pe}},2}}} \cr } } \right]\left[ {\matrix{ {{\bf{p}}{{\bf{e}}_1}} \cr {{\bf{p}}{{\bf{e}}_2}} \cr } } \right] + \left[ {\matrix{ {{{\bf{Z}}_{{\bf{s}},1}}} & 0 \cr 0 & {{{\bf{Z}}_{{\bf{s}},2}}} \cr } } \right]\left[ {\matrix{ {{{\bf{s}}_1}} \cr {{{\bf{s}}_2}} \cr } } \right] + \left[ {\matrix{ {{{\bf{e}}_1}} \cr {{{\bf{e}}_2}} \cr } } \right] \cr}$$

5 $$\:\left[\begin{array}{c}{\mathbf{c}}_{\mathbf{h}\mathbf{y}\mathbf{s},1}\\\:{\mathbf{c}}_{\mathbf{h}\mathbf{y}\mathbf{s},2}\end{array}\right]=\mathbf{N}\left(\left[\begin{array}{c}0\\\:0\end{array}\right],\left[\begin{array}{cc}{{\boldsymbol{\upsigma\:}}^{2}}_{\mathbf{h}\mathbf{y}\mathbf{s}1}&\:{\boldsymbol{\upsigma\:}}_{\mathbf{h}\mathbf{y}\mathbf{s}1,\mathbf{h}\mathbf{y}\mathbf{s}2}\\\:{\boldsymbol{\upsigma\:}}_{\mathbf{h}\mathbf{y}\mathbf{s}1,\mathbf{h}\mathbf{y}\mathbf{s}2}&\:{{\boldsymbol{\upsigma\:}}^{2}}_{\mathbf{h}\mathbf{y}\mathbf{s}2}\end{array}\right]\otimes\:\mathbf{I}\right)$$

6 $$\:\left[\begin{array}{c}{\mathbf{p}\mathbf{e}}_{1}\\\:{\mathbf{p}\mathbf{e}}_{2}\end{array}\right]=\mathbf{N}\left(\left[\begin{array}{c}0\\\:0\end{array}\right],\left[\begin{array}{cc}{{\boldsymbol{\upsigma\:}}^{2}}_{\mathbf{p}\mathbf{e}1}&\:{\boldsymbol{\upsigma\:}}_{\mathbf{p}\mathbf{e}1,\mathbf{p}\mathbf{e}2}\\\:{\boldsymbol{\upsigma\:}}_{\mathbf{p}\mathbf{e}1,\mathbf{p}\mathbf{e}2}&\:{{\boldsymbol{\upsigma\:}}^{2}}_{\mathbf{p}\mathbf{e}2}\end{array}\right]\otimes\:\mathbf{I}\right)$$

7 $$\:\left[\begin{array}{c}{\mathbf{s}}_{1}\\\:{\mathbf{s}}_{2}\end{array}\right]=\mathbf{N}\left(\left[\begin{array}{c}0\\\:0\end{array}\right],\left[\begin{array}{cc}{{\boldsymbol{\upsigma\:}}^{2}}_{\mathbf{a},\mathbf{s}1}&\:{\boldsymbol{\upsigma\:}}_{\mathbf{a},\mathbf{s}1\mathbf{s}2}\\\:{\boldsymbol{\upsigma\:}}_{\mathbf{a},\mathbf{s}1\mathbf{s}2}&\:{{\boldsymbol{\upsigma\:}}^{2}}_{\mathbf{a},\mathbf{s}2}\end{array}\right]\otimes\:\mathbf{A}\right)$$

8 $$\:\left[\begin{array}{c}{\mathbf{e}}_{1}\\\:{\mathbf{e}}_{2}\end{array}\right]=\mathbf{N}\left(\left[\begin{array}{c}0\\\:0\end{array}\right],\left[\begin{array}{cc}{{\boldsymbol{\upsigma\:}}^{2}}_{\mathbf{e}1}&\:0\\\:0&\:{{\boldsymbol{\upsigma\:}}^{2}}_{\mathbf{e}2}\end{array}\right]\otimes\:\mathbf{I}\right)$$

Where $\:{\mathbf{y}}_{1}$ and $\:{\mathbf{y}}_{2}$ were vectors of phenotypic observations for the two considered bins, **X** was the incidence matrix relating observations to fixed effects of each bin, **b** was the vector of fixed effects, **Z**_**hys, i**_ was the incidence matrix relating observations of bin *i* to the herd-year-season at farrowing, **c**_**hys, i**_ was the vector of bin *i* of the herd-year-season at farrowing effect; **Z**_**pe, i**_ was the incidence matrix relating observations of bin *i* to the permanent environmental effects, **pe**_**i**_ was the vector of bin *i* of permanent environmental effect; **Z**_**s, i**_ was the incidence matrix relating observations of bin *i* to the random sire genetic effects, **s**_**i**_ is the vector sire random effects for bin *i*-or the breeding values of the sires-, $\:\mathbf{e}$ is the vector of residuals,

In the distribution of the genetic effects, $\:{{\boldsymbol{\upsigma\:}}^{2}}_{\mathbf{s}1}$ was the variance of the sire effects for bin 1; $\:{{\boldsymbol{\upsigma\:}}^{2}}_{\mathbf{s}2}$ was the variance of the sire effects for bin 2; $\:{\boldsymbol{\upsigma\:}}_{\mathbf{s}1\mathbf{s}2}$ was the covariance of the sire effects between bins, $\:\otimes\:$ denoted the Kronecker product of matrices, and **A** was the matrix of the additive genetic relationships between sires, constructed using pedigree information. The residual variance–covariance matrix was constrained to be diagonal, assuming no residual covariance between bins.

- For NT:

9 $$\eqalign{ \left[ {\matrix{ {{{\bf{y}}_1}} \cr {{{\bf{y}}_2}} \cr } } \right] & = \left[ {\matrix{ {{{\bf{X}}_1}} & 0 \cr 0 & {{{\bf{X}}_2}} \cr } } \right]\left[ {\matrix{ {{{\bf{b}}_1}} \cr {{{\bf{b}}_2}} \cr } } \right] + \left[ {\matrix{ {{\bf{Zhysbirt}}{{\bf{h}}_1}} & 0 \cr 0 & {{\bf{Zhysbirt}}{{\bf{h}}_2}} \cr } } \right]\left[ {\matrix{ {{\bf{chysbirt}}{{\bf{h}}_1}} \cr {{\bf{chysbirt}}{{\bf{h}}_2}} \cr } } \right] \cr & + \left[ {\matrix{ {{\bf{Zd}}{{\bf{e}}_1}} & 0 \cr 0 & {{\bf{Zd}}{{\bf{e}}_2}} \cr } } \right]\left[ {\matrix{ {{\bf{d}}{{\bf{e}}_1}} \cr {{\bf{d}}{{\bf{e}}_2}} \cr } } \right] + \left[ {\matrix{ {{\bf{Z}}{{\bf{s}}_1}} & 0 \cr 0 & {{\bf{Z}}{{\bf{s}}_2}} \cr } } \right]\left[ {\matrix{ {{{\bf{s}}_1}} \cr {{{\bf{s}}_2}} \cr } } \right] + \left[ {\matrix{ {{{\bf{e}}_1}} \cr {{{\bf{e}}_2}} \cr } } \right] \cr}$$

Where $\:{\mathbf{y}}_{1}$ and $\:{\mathbf{y}}_{2}$ were vectors of phenotypic observations for each bin, **X** was the incidence matrix relating observations to fixed effects of each bin, **b** was the vector of fixed effects, **Z**_**hysbirth**,i_ was the incidence matrix relating observations of bin *i* to the herd-year-season at birth, **c**_**hysbirth, i**_ was the vector of bin *i* of the herd-year-season at birth effect; **Zs**_**i**_ was the incidence matrix relating observations of bin *i* to the random sire genetic effects, **s**_**i**_ is the vector sire random effects for bin *i*-or the breeding values of the sires-, $\:\boldsymbol{\upepsilon\:}$ is the vector of residuals. The distribution of the random effects were analogous to those of the traits NBA, TNB and NSBbc.

- For SLDG:

10 $$\eqalign{ \left[ {\matrix{ {{{\bf{y}}_1}} \cr {{{\bf{y}}_2}} \cr } } \right] & = \left[ {\matrix{ {{{\bf{X}}_1}} & 0 \cr 0 & {{{\bf{X}}_2}} \cr } } \right]\left[ {\matrix{ {{{\bf{b}}_1}} \cr {{{\bf{b}}_2}} \cr } } \right] + \left[ {\matrix{ {{{\bf{Z}}_{{\bf{pg}},1}}} & 0 \cr 0 & {{{\bf{Z}}_{{\bf{pg}},2}}} \cr } } \right]\left[ {\matrix{ {{\bf{cp}}{{\bf{g}}_1}} \cr {{\bf{cp}}{{\bf{g}}_2}} \cr } } \right] \cr & + \left[ {\matrix{ {{{\bf{Z}}_{{\bf{litter}},1}}} & 0 \cr 0 & {{{\bf{Z}}_{{\bf{litter}},2}}} \cr } } \right]\left[ {\matrix{ {{\bf{litte}}{{\bf{r}}_1}} \cr {{\bf{litte}}{{\bf{r}}_2}} \cr } } \right] + \left[ {\matrix{ {{{\bf{Z}}_{{\bf{s}},1}}} & 0 \cr 0 & {{{\bf{Z}}_{{\bf{s}},2}}} \cr } } \right]\left[ {\matrix{ {{{\bf{s}}_1}} \cr {{{\bf{s}}_2}} \cr } } \right] + \left[ {\matrix{ {{{\bf{e}}_1}} \cr {{{\bf{e}}_2}} \cr } } \right] \cr}$$

Where $\:{\mathbf{y}}_{1}$ and $\:{\mathbf{y}}_{2}$ were vectors of phenotypic observations for each bin, $\:\mathbf{X}$ was the incidence matrix relating observations to fixed effects of each bin, **b** was the vector of fixed effects, **Z**_**pg.i**_ was the incidence matrix relating observations of bin *i* to the pen group effect, **cpg**_**i**_ was the vector of bin *i* of the pen group effect; **Z**_**litter, i**_ was the incidence matrix relating observations of bin *i* to the litter effect, **litter**_**i**_ was the vector of bin *i* of the litter effect; **Z**_**s, i**_ was the incidence matrix relating observations of bin *i* to the random sire genetic effects, **s**_**i**_ is the vector sire random effects for bin *i-*or the breeding values of the sires-, $\:\mathbf{e}$ is the vector of residuals. The distribution of the random effects were similar to those of the other traits. All bivariate models between bins converged for NBA, and 9 out of 10 models converged for TNB and NSBbc. For NT and SLDG in both lines, fewer than 4 out of 10 models converged, so we only report the results for traits with the majority of models converging.

### Random regression models

In addition to the univariate and bivariate analysis, we used random regression models (RRMs) with Legendre polynomials to estimate the trajectory of the $\:{\sigma\:}_{a,sire}^{2}$ as a function of ACCs. To our knowledge, RRMs have not been previously used to model variance across ACC observations. We chose this approach because it allows sire breeding values to be modelled along a continuous ACCs gradient by estimating random regression coefficients for each sire [21]. As ACC represents the number of crossovers produced by a parent and transmitted to an offspring, each offspring has only a single ACC record in its lifetime coming from each progenitor, making longitudinal analyses impossible. In contrast to the number of crossovers that an individual received, there can be multiple observations of the number of crossovers that a sire transmits to its offspring, which makes it possible to model the breeding values of the sires as a function of the number of crossovers that the offspring received. The resulting random regression curves can then be used to derive estimates of $\:{\sigma\:}_{a,sire}^{2}$ across the ACCs gradient. This step will be explained later in more detail. In the RRMs, phenotypic records of reproduction traits and number of teats from female offspring were used to estimate sire additive genetic effects. This model was applied for each breeding goal trait separately. For the trait SLDG, phenotypic records from both female and male offspring were used.

The used RRM model was the following:

For NBA and TNB:

11 $$\:\mathbf{y}=\mathbf{X}\mathbf{b}+{\mathbf{Z}}_{\mathbf{h}\mathbf{y}\mathbf{s}}\mathbf{c}\mathbf{h}\mathbf{y}\mathbf{s}+{\mathbf{Z}}_{\mathbf{p}\mathbf{e}}\mathbf{p}\mathbf{e}+{\mathbf{Z}}_{0}{\boldsymbol{a}}_{0}+{\mathbf{Z}}_{1}{\boldsymbol{a}}_{1}+{\mathbf{Z}}_{2}{\boldsymbol{a}}_{2}+{\mathbf{Z}}_{3}{\boldsymbol{a}}_{3}+\mathbf{e}$$

For NSBbc:

12 $$\:\mathbf{y}=\mathbf{X}\mathbf{b}+{\mathbf{Z}}_{\mathbf{h}\mathbf{y}\mathbf{s}}\mathbf{c}\mathbf{h}\mathbf{y}\mathbf{s}+{\mathbf{Z}}_{\mathbf{p}\mathbf{e}}\mathbf{p}\mathbf{e}+{\mathbf{Z}}_{0}{\boldsymbol{a}}_{0}+\mathbf{e}$$

For NT:

13 $$\:\mathbf{y}=\mathbf{X}\mathbf{b}+{\mathbf{Z}}_{\mathbf{h}\mathbf{y}\mathbf{s}\mathbf{b}\mathbf{i}\mathbf{r}\mathbf{t}\mathbf{h}}\mathbf{c}\mathbf{h}\mathbf{y}\mathbf{s}\mathbf{b}\mathbf{i}\mathbf{r}\mathbf{t}\mathbf{h}+{\mathbf{Z}}_{\mathbf{d}\mathbf{e}}\mathbf{d}\mathbf{e}+{\mathbf{Z}}_{0}{\boldsymbol{a}}_{0}+\mathbf{e}$$

For SLDG:

14 $$\:\mathbf{y}=\mathbf{X}\mathbf{b}+{\mathbf{Z}}_{\mathbf{p}\mathbf{g}}\mathbf{p}\mathbf{g}+{\mathbf{Z}}_{\mathbf{l}\mathbf{i}\mathbf{t}\mathbf{t}\mathbf{e}\mathbf{r}}\mathbf{l}\mathbf{i}\mathbf{t}\mathbf{t}\mathbf{e}\mathbf{r}+{\mathbf{Z}}_{0}{\boldsymbol{a}}_{0}+\mathbf{e}$$

Where X, b, $\:{\mathbf{Z}}_{\mathbf{h}\mathbf{y}\mathbf{s}}$, $\:\mathbf{c}\mathbf{h}\mathbf{y}\mathbf{s},$ $\:{\mathbf{Z}}_{\mathbf{p}\mathbf{e}}$, $\:\mathbf{p}\mathbf{e}$, $\:{\mathbf{Z}}_{\mathbf{h}\mathbf{y}\mathbf{s}\mathbf{b}\mathbf{i}\mathbf{r}\mathbf{t}\mathbf{h}}$, $\:\mathbf{c}\mathbf{h}\mathbf{y}\mathbf{s}\mathbf{b}\mathbf{i}\mathbf{r}\mathbf{t}\mathbf{h}$, $\:{\mathbf{Z}}_{\mathbf{p}\mathbf{g}}$, $\:\mathbf{p}\mathbf{g}$, $\:{\mathbf{Z}}_{\mathbf{l}\mathbf{i}\mathbf{t}\mathbf{t}\mathbf{e}\mathbf{r}}$, $\:\mathbf{l}\mathbf{i}\mathbf{t}\mathbf{t}\mathbf{e}\mathbf{r}$ and e were similarly defined as in the univariate and bivariate models; $\:{\boldsymbol{a}}_{0}$, $\:{\boldsymbol{a}}_{1}$, $\:{\boldsymbol{a}}_{2}$, $\:{\boldsymbol{a}}_{3}$ are vectors of the sires’ random regression coefficients associated with the Legendre Polynomials of the standardised covariate for the intercept, slope, quadratic and cubic terms; $\:{\mathbf{Z}}_{0}$ is the incidence matrix relating observations to sires, while, $\:{\mathbf{Z}}_{1},{\mathbf{Z}}_{2},{\mathbf{Z}}_{3}$ are incidence matrices scaled by the first, second, and third order Legendre polynomial of the standardised covariate, respectively, linking observations to sire-specific random regression coefficients. Likewise for the univariate and bivariate models, here we also included ACCs as a fixed effect in the model, so the random regression coefficients represent deviations from the fixed curve for each level of ACC.

Random regression models (RRM) allow the estimation of a $\:\left(q+1\right)$ x $\:\left(q+1\right)$ variance-covariance matrix $\:\mathbf{G}$, with * q* being the order of the polynomial, which contains the variances and covariances of the random regression coefficients:

For NBA and TNB, the $\:\mathbf{G}$ matrix is:

15 $$\:\mathbf{G}=\:\left[\begin{array}{cccc}{\mathrm{V}\mathrm{a}\mathrm{r}(a}_{0})&\:\mathrm{C}\mathrm{o}\mathrm{v}({a}_{0},{a}_{1})&\:\mathrm{C}\mathrm{o}\mathrm{v}({a}_{0},{a}_{2})&\:\mathrm{C}\mathrm{o}\mathrm{v}({a}_{0},{a}_{3})\\\:\mathrm{C}\mathrm{o}\mathrm{v}({a}_{1},{a}_{0})&\:{\mathrm{V}\mathrm{a}\mathrm{r}(a}_{1})&\:\mathrm{C}\mathrm{o}\mathrm{v}({a}_{1},{a}_{2})&\:\mathrm{C}\mathrm{o}\mathrm{v}({a}_{1},{a}_{3})\\\:\mathrm{C}\mathrm{o}\mathrm{v}({a}_{2},{a}_{0})&\:\mathrm{C}\mathrm{o}\mathrm{v}({a}_{2},{a}_{1})&\:{\mathrm{V}\mathrm{a}\mathrm{r}(a}_{2})&\:\mathrm{C}\mathrm{o}\mathrm{v}({a}_{2},{a}_{3})\\\:\mathrm{C}\mathrm{o}\mathrm{v}({a}_{3},{a}_{0})&\:\mathrm{C}\mathrm{o}\mathrm{v}({a}_{3},{a}_{1})&\:\mathrm{C}\mathrm{o}\mathrm{v}({a}_{3},{a}_{2})&\:{\mathrm{V}\mathrm{a}\mathrm{r}(a}_{3})\end{array}\right]$$

For NSBbc, NT and SLDG, the $\:\mathbf{G}$ matrix is:

16 $$\:\mathbf{G}=\:\left[{\mathrm{V}\mathrm{a}\mathrm{r}(a}_{0})\right]$$

To then obtain $\:{\sigma\:}_{a,sire}^{2}$ as a function of ACCs, we used:

17 $$\:\mathbf{V}=\:\boldsymbol{\upphi\:}\mathbf{G}{\boldsymbol{\upphi\:}}^{{\prime\:}}$$

Where $\:\boldsymbol{\upphi\:}\:$is a matrix of dimension $\:t\:\mathrm{x}\:(q+1)$, with *t* being the number of ACCs levels being equal to 27 (TNB and NT), 28 (NBA), 29 (NSBbc and SLDG for the boar line) and 30 (SLDG for the sow line). Each row of $\:\boldsymbol{\upphi\:}$ contains the values of the Legendre polynomials at a given standardized ACC level. Standardization of ACCs rescales the covariate to the interval [− 1,1] using: $\:{ACC}_{i}=-1+2\left(\frac{{ACCs}_{i}-A{CCs}_{min}}{{ACCs}_{max}-{ACCs}_{min}}\right)$. This standardization is performed automatically by ASReml when modelling RRM using Legendre polynomials.$\:\mathbf{V}$ is a *t* x *t* matrix, where diagonal elements represent the variances for each ACCs and off-diagonal elements their covariances.

In our analyses, we increased the order of the polynomial until log-likelihood ratio tests indicated no further significant improvement in model fit or until the model failed to converge [22], which was the case for TNB and NBA. Results are presented only for the polynomial order which fitted the data best. For NBA and TNB, this was the 4th order and for NT, NSBbc and SLDG in both lines this was the zero polynomial order.

## Results

### Univariate and bivariate models

**Fig. 1:**
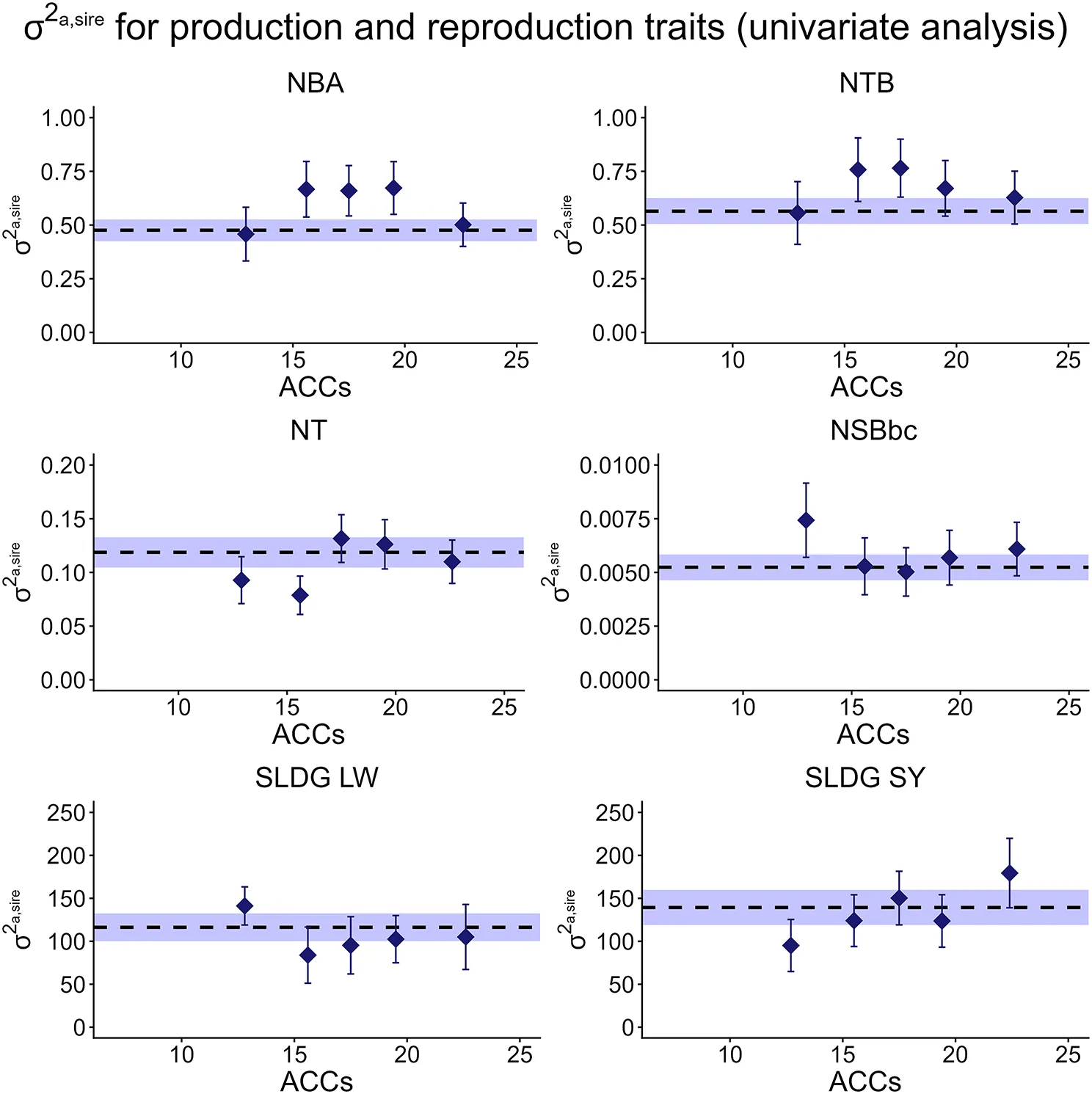
$\:{\widehat{\boldsymbol{\sigma\:}}}_{\boldsymbol{a},\boldsymbol{s}\boldsymbol{i}\boldsymbol{r}\boldsymbol{e}}^{2}$ of reproduction and production traits estimated using univariate models for each ACC-defined bin. Each bin is positioned at the x-value corresponding to the mean ACCs within that bin. Error bars represent the standard error. The dotted line represents the estimated $\:{\sigma\:}_{a,sire}^{2}$ without accounting for bins and the shaded area represents its standard error

The overall $\:{\widehat{\sigma\:}}_{a,sire}^{2}$ for each breeding goal trait estimated using the data of all five ACCs bins together, as well as within each bin, is presented in Table 4. Results indicate that for all traits, the 95% CI of the overall $\:{\widehat{\sigma\:}}_{a,sire}^{2}$ of the trait overlapped with estimates for each of the bins, even for the bin with the highest $\:{\widehat{\sigma\:}}_{a,sire}^{2}$, indicating that the $\:{\widehat{\sigma\:}}_{a,sire}^{2}\:$of the analyzed traits is not significantly affected by the observed number of crossovers. Despite the lack of significant difference, the overall trends in $\:{\widehat{\sigma\:}}_{a,sire}^{2}$ across ACCs ranges suggested that $\:{\widehat{\sigma\:}}_{a,sire}^{2}$ was maximised at intermediate ACCs ranges for NBA, TNB and NT, at lower ACCs ranges for NSBbc, and towards higher ACCs ranges for SLDG. In addition, the trends in the results indicate that for NBA and TNB, $\:{\widehat{\sigma\:}}_{a,sire}^{2}$ estimates of the separate bins were higher than the $\:{\widehat{\sigma\:}}_{a,sire}^{2}$ obtained using the full dataset except for the first bin (Table 4; Fig. 1). For NT, only bins 3 and 4 showed higher$\:\:{\widehat{\sigma\:}}_{a,sire}^{2}$ than the estimate based on all data (Table 4; Fig. 1). For NSBbc, results indicated a trend of higher $\:{\widehat{\sigma\:}}_{a,sire}^{2}$ at lower and higher ACCs values, with bins 1, 4 and 5 showing higher estimates than the full-dataset estimate (Table 4; Fig. 1). For SLDG, in the sow line, $\:{\widehat{\sigma\:}}_{a,sire}^{2}$ estimates were lower than the full-dataset estimate in all bins except the first, while in the boar line this was the case for all bins except the third and fifth (Fig. 1). Results of the estimated heritabilities (*h*^*2*^) indicated similar patterns to those of the $\:{\widehat{\sigma\:}}_{a,sire}^{2}$ [Additional file 2, Table S1].

**Fig. 2:**
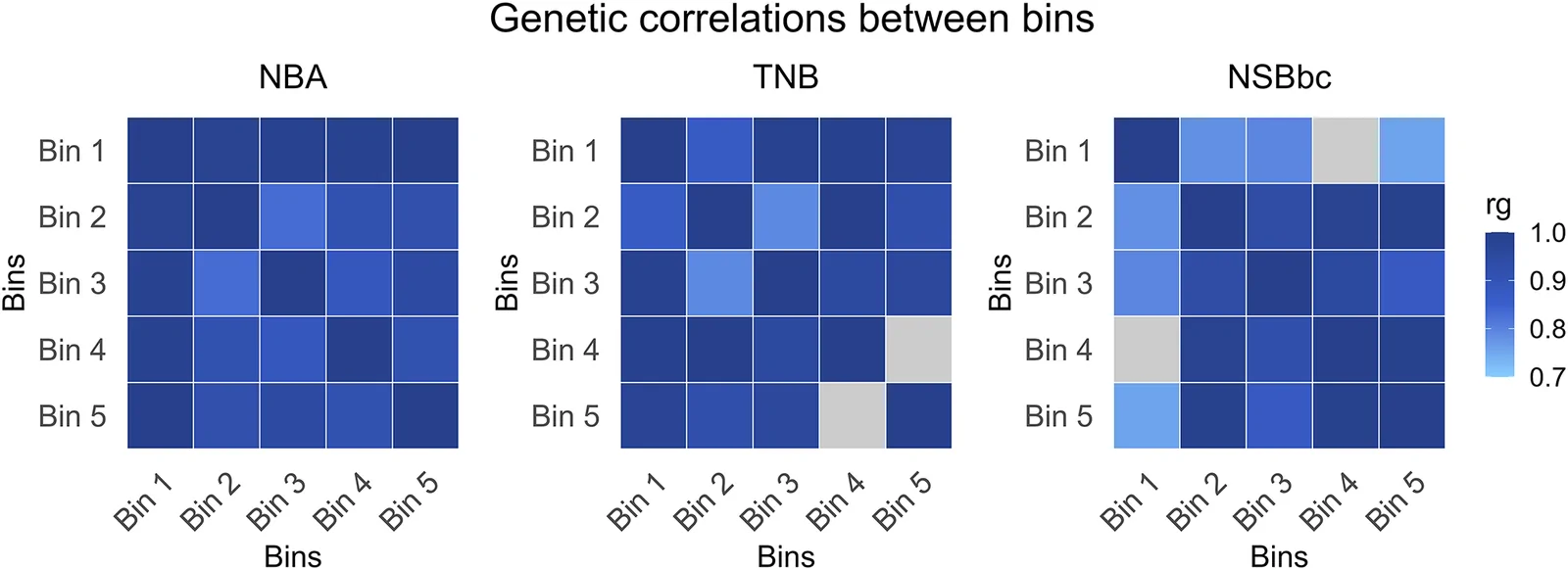
Genetic correlations between bins, for each trait using bivariate models. Correlations shown in grey correspond to bivariate models between bins that did not converge

Moreover, the 95% confidence intervals for the differences in $\:{\widehat{\sigma\:}}_{a,sire}^{2}$ between consecutive bins also overlapped, except for bins 2 and 3 in NT. These results indicate that the estimated $\:{\sigma\:}_{a,sire}^{2}$ didn’t show significant differences between consecutive ACCs bins (Table 4). Overall, the bins with the lowest and highest $\:{\widehat{\sigma\:}}_{a,sire}^{2}$ showed an average difference of 57% across all traits.

**Table 4:** Estimated sire additive genetic variance ($\:{\widehat{\boldsymbol{\sigma\:}}}_{\boldsymbol{a},\boldsymbol{s}\boldsymbol{i}\boldsymbol{r}\boldsymbol{e}}^{2})$and corresponding standard error (SE), shown for each trait and bin from the univariate and bivariate analyses

Trait	$\:{\sigma\:}_{a,sire}^{2}$(SE) from univariate models using all the dataset				
NBA	0.48 (0.06)				
TNB	0.56 (0.07)				
NT	0.12 (0.01)				
NSBbc	0.005 (0.001)				
SLDG (sow line)	116.29 (15.97)				
SLDG (boar line)	139.36 (20.43)				
	$\:{\sigma\:}_{a,sire}^{2}$(SE) from univariate models using bins				
	Bin 1	Bin 2	Bin 3	Bin 4	Bin 5
NBA	0.46 (0.12)	0.67 (0.13)	0.66 (0.12)	0.67 (0.12)	0.50 (0.10)
TNB	0.56 (0.14)	0.76 (0.14)	0.76 (0.13)	0.67 (0.12)	0.63 (0.12)
NT	0.09 (0.02)	0.08 (0.02)	0.13 (0.02)	0.13 (0.02)	0.11 (0.02)
NSBbc	0.007 (0.001)	0.005 (0.001)	0.005 (0.001)	0.006 (0.001)	0.006 (0.001)
SLDG (sow line)	141.129 (32.9)	83.9 (22.1)	95.3 (21.1)	102.5 (22.8)	105.0 (22.2)
SLDG (boar line)	95.1 (30.3)	124.0 (30.1)	150.3 (31.2)	123.6 (30.5)	179.4 (40.4)
	$\:{\sigma\:}_{a,sire}^{2}\:$(SE) from bivariate models using bins^a^				
NBA	0.43 (0.11)	0.62 (0.12)	0.63 (0.11)	0.62 (0.11)	0.49 (0.09)
TNB	0.53 (0.13)	0.71 (0.14)	0.72 (0.16)	0.58 (0.11)	0.62 (0.12)
NSBbc	0.007 (0.001)	0.005 (0.001)	0.005 (0.001)	0.006 (0.001)	0.006 (0.001)

^a^The$\:{\widehat{\sigma\:}}_{a,sire}^{2}$from each bin corresponds to the average of the variances obtained for that bin across all analysis including that bin

For each analysed trait, $\:{\widehat{\sigma\:}}_{a,sire}^{2}$ from the bivariate models exhibited patterns similar to those observed in the univariate analyses (Table 4). Additionally, within each trait, genetic correlations among bins were high (between 0.79 and 0.99), except for the correlations between bin 1 and the others within NSBbc, which were below 0.8 [Additional File 1, Figure S2 and Additional File 2, Table S2] (Fig. 2).

The results for the univariate dam models are in [Additional file 1, Figure S1 and Additional file 2]. Overall, sires and dams showed similar patterns for NBA and TNB, where $\hat{\sigma}_{a,sire}^{2}$ and $\:{\widehat{\sigma\:}}_{a,dam}^{2}$ is maximised at intermediate ACC. In contrast, the remaining traits exhibited distinct patterns between sires and dams.

### Random regression models

To explore the continuous trajectory of $\:{\sigma\:}_{a,sire}^{2}$ across ACCs, we fitted random regression models using Legendre polynomials of increasing order and compared their goodness of fit to determine whether higher-order terms significantly improved the model. For TNB and NBA, the analysis indicated that the 4th-order polynomial provided a significantly better fit than lower-degree models [Additional file 2, Table S5]. For both those traits, increasing the order of the polynomial to five resulted in non-convergence, as the estimated **G** matrix was not positive definite. For NSBbc, NT and SLDG, the model didn’t significantly improve with fitting higher polynomial orders than a 0th-order [Additional file 2, Table S5].

**Fig. 3:**
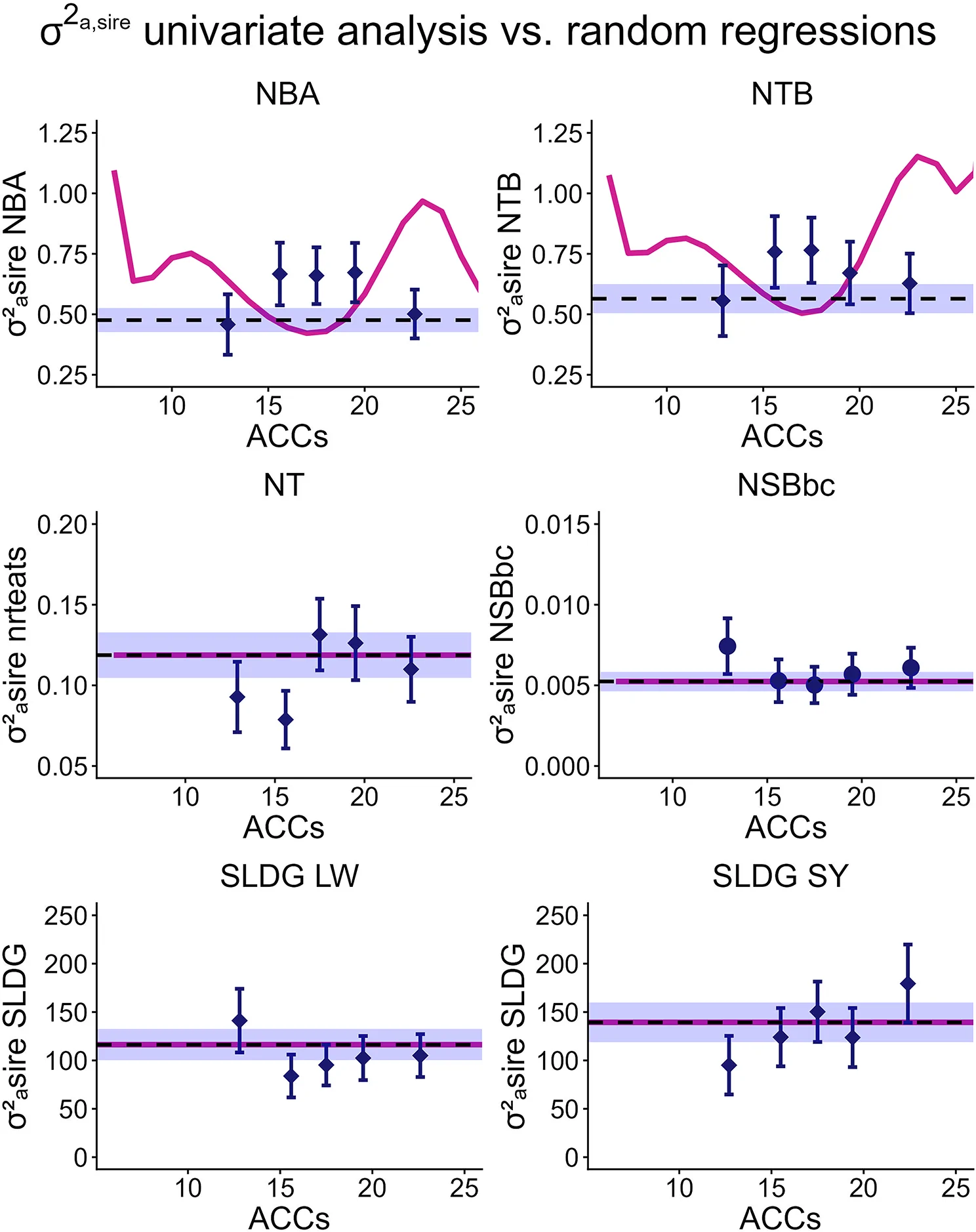
$\:{\widehat{\boldsymbol{\sigma\:}}}_{\boldsymbol{a},\boldsymbol{s}\boldsymbol{i}\boldsymbol{r}\boldsymbol{e}}^{2}$ from univariate models versus random regression models. Each bin of univariate analysis is positioned at the x-value corresponding to the mean ACCs within that bin. Error bars represent the standard error. The dotted line represents the $\:{\widehat{\sigma\:}}_{a,sire}^{2}$ without accounting for bins and the shaded area represents its standard error

Because the best-fitting polynomial order differed among traits, estimates of $\:{\sigma\:}_{a,sire}^{2}$ from the RRMs revealed different patterns among traits (Fig. 3). Overall, for the traits NSBbc, NT and SLDG, $\:{\widehat{\sigma\:}}_{a,sire}^{2}$ remained constant across the range of ACCs. In contrast, for NBA and TNB, $\:{\widehat{\sigma\:}}_{a,sire}^{2}$ showed changed across the range of the covariate. For NBA and TNB, the obtained function for estimating $\:{\widehat{\sigma\:}}_{a,sire}^{2}$ as function of the ACCs suggests that middle ACCs ranges minimize $\:{\widehat{\sigma\:}}_{a,sire}^{2}$while both lower and higher ACCs values maximise it. Note that Fig. 3 only includes ACCs values until ACCs = 25 because the majority of the observations are ≤ 25. Values > 25 were rare ( < ~ 1.7%), making results unreliable. For both NBA and TNB, the $\:{\widehat{\sigma\:}}_{a,sire}^{2}\:$increased sharply at extreme ACCs values, leading to biologically implausible estimates and making visualization of the results impractical [Additional file 1, Figure S2]. Moreover, the results show that the pattern observed with the RRM is opposite to that obtained from the univariate models.

For comparison, the results are presented together with those obtained from the univariate analyses using bins of observations.

## Discussion

In this study, we aimed to study the link between ACC and the additive genetic variance using empirical data for different reproduction and production traits in pigs. This was done by estimating the $\:{\sigma\:}_{a,sire}^{2}$ to use it as a proxy for the total $\:{\sigma\:}_{a}^{2}$, considering bins of individuals in the population defined by the number of paternal autosomal crossovers they inherited. Results indicated that there were no significant differences in $\:{\widehat{\sigma\:}}_{a,sire}^{2}\:$between bins. However, trends in $\:{\widehat{\sigma\:}}_{a,sire}^{2}$ were apparent and differed between the analysed traits. For three of the traits (NBA, TNB and NT), the $\:{\widehat{\sigma\:}}_{a,sire}^{2}$was maximised within middle ACCs ranges, while for the other traits it was either maximised towards lower or higher ranges.

### Patterns of $\:{\widehat{\boldsymbol{\sigma\:}}}_{\boldsymbol{a},\boldsymbol{s}\boldsymbol{i}\boldsymbol{r}\boldsymbol{e}}^{2}\:$in RRM, univariate and bivariate models

In the random regression analyses, results for $\:{\widehat{\sigma\:}}_{a,sire}^{2}\:$increased sharply at extreme high ACCs values, leading to biologically implausible estimates [Additional file 1, Figure S2]. This pattern towards extreme values of the covariate was previously observed in several studies using random regression models [22, 23]. Furthermore, we found clear discrepancies between the trend of $\:{\widehat{\sigma\:}}_{a,sire}^{2}$ between the RRM and univariate models for all traits. This discrepancy may arise because in the RRM $\:{\widehat{\sigma\:}}_{a,sire}^{2}$ at each ACC depends on the covariance structure of the random regression coefficients (**G**), which can lead to extreme variance estimates, particularly at the covariate extremes. In addition, ACC itself is a partially heritable trait and therefore does not constitute a strictly non-genetic gradient. Previous studies [24, 25] have shown that when the covariate shares genetic determinants with the response trait, genotype–covariate correlation may be partially confounded with genotype–covariate interaction if not explicitly modelled, potentially leading to difficult-to-interpret variance component estimates. Although the heritability of ACC previously reported in other studies was low (ranging between 0.07 and 0.11 in females and between 0.04 and 0.05 in males) [7, 14], suggesting that any overlap between the additive genetic effects underlying ACC and the fertility traits is likely to be limited, this source of confounding cannot be completely excluded. Due to this parametric nature of the random regression models, the underlying pattern of $\:{\widehat{\sigma\:}}_{a,sire}^{2}$ may not be fully captured. In contrast, univariate models provide independent $\:{\widehat{\sigma\:}}_{a,sire}^{2}$ for each ACCs-defined bin. Moreover, while multivariate models could not be reliably fitted due to convergence issues, the univariate estimates offer a robust and interpretable approximation of the pattern of $\:{\widehat{\sigma\:}}_{a,sire}^{2}$ across ACCs bins. For all these reasons, we placed greater confidence in the patterns inferred from the univariate results than those obtained from the RRM.

Our results showed that for all traits, the 95% CI of the overall $\:{\widehat{\sigma\:}}_{a,sire}^{2}$ of the trait overlapped with the $\:{\widehat{\sigma\:}}_{a,sire}^{2}$ estimated for each bin, indicating that the $\:{\widehat{\sigma\:}}_{a,sire}^{2}\:$of the analyzed traits is not significantly affected by the observed number of crossovers. However, the traits differed in the trend of $\:{\widehat{\sigma\:}}_{a,sire}^{2}$ across the bins of ACC. This may indicate that the potential utility of crossovers in breaking up the link between nearby loci may depend on the architecture of the trait, such as its degree of polygenicity and the genomic distribution of the polygenes affecting the trait. For example, in the most frequently observed pattern, intermediate ACCs appeared to maximise $\:{\widehat{\sigma\:}}_{a,sire}^{2}$ (traits NT, TNB, and NBA), suggesting a potential optimal effect of ACCs rather than a continuous increase in variance with increasing ACCs as predicted by our hypothesis. One possible explanation for this trend may be that a low number of crossovers may be insufficient to break the link between loci and generate enough novel allele combinations. As the number of crossovers increases, the reshuffling of alleles across loci may produce more diverse genetic combinations, thereby increasing the estimated $\:{\widehat{\sigma\:}}_{a,sire}^{2}$. However, at very high ACCs, additional crossover events could disrupt more extreme allele combinations, resulting in no further increase or even a reduction in $\:{\widehat{\sigma\:}}_{a,sire}^{2}$. In addition, for those traits in which the trend of $\:{\widehat{\sigma\:}}_{a,sire}^{2}$ maximised at higher ACCs ranges (SLDG in both pig lines), this might reflect the need for more crossovers to break the link between nearby loci, thereby creating on average more extreme haplotypes, and as a consequence the $\:{\widehat{\sigma\:}}_{a,sire}^{2}$ increases. One possible explanation might be that favourable and unfavourable alleles are distributed across homologous paternal chromosomes, allowing crossovers to generate allele combinations with more extreme genetic effects. In contrast, for the trait NSBbc, in which the trend of $\:{\widehat{\sigma\:}}_{a,sire}^{2}$ was maximised towards lower ACCs, the opposite mechanism may occur. If paternal haplotypes are already highly differentiated before meiotic recombination, a high number of crossovers may facilitate the exchange of alleles between homologous chromosomes, producing less haplotypes with extreme allele combinations. As a result, the genetic contrast among haplotypes may be reduced, leading to lower $\:{\widehat{\sigma\:}}_{a,sire}^{2}$ at higher ACC levels.

For NT, the trend that intermediate crossover bins showed a higher $\:{\widehat{\sigma\:}}_{a,sire}^{2}$ was slightly clearer than for the other traits. Here, $\:{\widehat{\sigma\:}}_{a,sire}^{2}\:$increased significantly between the middle bins (particularly between bins 2 and 3). This clearer pattern could be a result of the previously reported higher heritability of NT [26, 27], which was observed in our study [Additional file 2, Table S1]. This is because differences in $\:{\widehat{\sigma\:}}_{a,sire}^{2}\:$across crossover bins might become more detectable when additive genetic effects account for a larger proportion of the phenotypic variance. Overall, these patterns suggest that the effect of crossover events on $\:{\widehat{\sigma\:}}_{a,sire}^{2}$ may depend on the genetic architecture of the trait.

Our results obtained with the bivariate models including two bins may suggest heterogeneity in the genetic architecture of number of stillborn piglets (NSBbc) as a function of the ACCs inherited. For this trait, the observed $\:{\widehat{\sigma\:}}_{a,sire}^{2}$ was highest at the bin with the lowest ACC. This bin also showed relatively lower genetic correlations with the other bins (ranging from 0.75 to 0.80), while the remaining bins were consistently highly correlated, i.e. having correlations greater than 0.88 [Additional file 1, Figure S2 and Additional file 2, Table S2]. This pattern was not observed for the other traits analysed, in which genetic correlations between bins were close to 1, suggesting a specific relationship between ACCs and NSBbc. Previous studies have highlighted the importance of meiotic crossovers for chromosome segregation and fertility performance [28], although studies directly investigating the relationship between recombination and fertility-related traits remain relatively limited. Nevertheless, several studies in humans and one study in pigs have suggested that lower crossover counts or reduced recombination levels are associated with an increased risk of meiotic errors and poorer reproductive outcomes [29–33]. Consistent with these observations, experimental studies in mice have shown that disruptions in crossover formation or regulation impair chromosome segregation, increase meiotic abnormalities, and compromise fertility [34]. Similarly, studies in Drosophila have demonstrated that altered crossover patterns increase the risk of meiotic non-disjunction [35]. Based on those findings, we expected a higher phenotypic mean for NSBbc for the low-ACCs bin. However, the observed means did not appear to differ across ACCs bins [Additional file 2, Table S6], suggesting that the potential relationship between ACCs and NSBbc may be more evident at the genetic level than at the phenotypic level. Moreover, we also evaluated whether the mean number of crossovers transmitted by a dam was associated with her own number of stillborn piglets. However, no evidence of an association was observed in the subset of dams with available own fertility records (results not shown). Although this analysis addresses the biological relationship between crossover count and fertility performance more directly, the analysis was based on 1,847 out of the total 4,323 dams, as those ones contained both ACCd and own stillborn. Future research may be valuable to further investigate this potential relationship and the mechanisms behind it.

Lastly, the patterns of $\:{\widehat{\sigma\:}}_{a,sire}^{2}$ and $\:{\widehat{\sigma\:}}_{a,dam}^{2}$ obtained using bins across ACC in the univariate analyses showed similar trends for NBA and TNB, but not for the remaining traits [Additional file 1, Figure S1 and Additional file 2, Table S4]. These differences may be related to the presence of heterochiasmy in pigs [7, 15, 36], as well as to sex-specific variation in the recombination landscape, as reported in previous studies [14, 37]. For example, in this study, the ranges of ACC ranged between 5 and 34 in paternal gametes and between 8 and 47 in maternal gametes [Additional file 2, Table S2 and Additional file 2, Table S5]. Such differences in both the total number of autosomal crossovers may influence how crossovers potentially break the link between loci, possibly leading to the observed discrepancies between the patterns of $\:{\widehat{\sigma\:}}_{a,sire}^{2}$ and $\:{\widehat{\sigma\:}}_{a,dam}^{2}$ across bins. Moreover, in addition to differences in the number of crossovers, sex-specific differences in the recombination landscape between sires and dams [7, 14] may also influence the patterns of $\:{\widehat{\sigma\:}}_{a,sire}^{2}$ and $\:{\widehat{\sigma\:}}_{a,dam}^{2}$, thereby contributing to the observed discrepancies between them. In addition, although paternal and maternal crossovers are independent, and therefore offspring observations may be allocated to different ACC bins in the sire and dam models, differences in bin composition do not by themselves explain the contrasting patterns of $\:{\widehat{\sigma\:}}_{a,sire}^{2}$ and $\:{\widehat{\sigma\:}}_{a,dam}^{2}$. Each model estimates the genetic contribution of the focal parent (sire or dam), while genetic variation not captured by this contribution is included in the residual variance. Consistent with this expectation, we performed an additional analysis which showed that transmitted paternal and maternal crossover counts were essentially uncorrelated (*r* ≈ 0). Therefore, the ACC of the non-focal parent is expected to be randomly distributed across bins of the focal parent and is unlikely to generate systematic differences in additive genetic variance across those ACC classes. Furthermore, although $\:{\widehat{\sigma\:}}_{a,sire}^{2}$ and $\:{\widehat{\sigma\:}}_{a,dam}^{2}$ are theoretically expected to be equal, as both represent ¼ of the total additive genetic variance, slight differences were observed in our results [Additional file 2, Table S1 and Additional file 2, Table S4]. This discrepancy may result from partial confounding between maternal permanent environmental effects and additive genetic effects in the dam models, which should be considered when interpreting the results [38]. Our results show that for all the NBA, TNB, NSBbc and NT, $\:{\widehat{\sigma\:}}_{pe}^{2}$ are consistently higher in the sire models compared to the dam models [Additional file 2, Table S1 and Additional file 2, Table S4]. Given that the dam genetic effect and the permanent environmental effect are both linked to the dam, albeit assuming different variance structures, the dam model may have some identifiability issues to estimate the (dam) genetic variance, while this is not the case for the genetic effects estimated in the sire model. As a consequence, we have greater confidence in the estimated heritabilities for the sire models, and the higher estimates for the dam models suggest that those might be slightly overestimated.

### Potential use of ACC in animal breeding

Previous studies suggested that selection for the number of autosomal crossovers in pigs may have limited potential application, due to the low heritability of the trait and physiological constraints associated with substantially increasing ACC [5, 7, 14]. A previous simulation study indicated that achieving noticeable genetic gain for breeding goal traits due to a higher recombination rate would require a 10- to 20- fold increase in recombination rate, which may not be biologically feasible [5]. In the present study, we explored the potential role of crossovers in animal breeding through their effect on the additive genetic variance of breeding goal traits by using empirical data. Our results showed that, for most of the traits, the trend in $\:{\widehat{\sigma\:}}_{a,sire}^{2}$ was not maximised at higher crossover ranges, which suggests that large increases in recombination rate may not be effective to influence $\:{\widehat{\sigma\:}}_{a,sire}^{2}$. In addition, by enabling the reshuffling of alleles, crossovers may generate new combinations of alleles that could modify the $\:{\widehat{\sigma\:}}_{a,sire}^{2}$ of a trait. The magnitude and direction of this effect, however, may depend on the genetic architecture of each specific breeding goal trait.

Although our results do not provide conclusive evidence that $\:{\widehat{\sigma\:}}_{a,sire}^{2}$ changes with ACCs, as the 95% confidence intervals of consecutive bins largely overlap, we observed an average difference of ~ 57% between the bins with the lowest and highest $\:{\widehat{\sigma\:}}_{a,sire}^{2}$. These differences could still be relevant from an animal breeding perspective, as this might indicate a potential use of ACC in releasing $\:{\widehat{\sigma\:}}_{a,sire}^{2}$ in breeding goal traits by optimising ACC within the currently observed range. Hence, from an animal breeding perspective, and considering the observed differences, selecting for intermediate ACC ranges may be advantageous for reproductive traits, making this strategy particularly relevant for the sow line, which is primarily bred for reproductive performance. In contrast, selecting for higher ACC ranges may be more favourable for production traits and therefore more relevant for the boar line, which is mainly bred for production performance, although only one trait was included in the analysis for this line.

Studies have reported that the absence or a very low number of crossovers can be associated to problems during meiotic segregation of homologous chromosomes [39], leading to non-disjunction and aneuploid gametes [29, 30, 32], which can reduce fertility performance [31, 33]. Also, a lower ACC can be associated to increased expression of deleterious alleles, which can have a negative impact on fertility performance [40]. Conversely, very high recombination rates may increase deleterious mutations and recombination load, disrupting favourable allele combinations [2]. Altogether, these previous findings suggest biological constraints associated to both low and high ACC, possibly implying that an intermediate level of ACC is optimal for fertility. Consistent with this, our results show that the most frequent observed trend of $\:{\widehat{\sigma\:}}_{a,sire}^{2}$ across ACCs levels is that intermediate ACCs maximises $\:{\widehat{\sigma\:}}_{a,sire}^{2}$ for reproduction traits, which might benefit breeding programs by enabling faster response to selection in breeding goal traits such as NBA, TNB and NSBbc. This means that if ACC is considered in breeding programs, its magnitude should be carefully monitored to prevent the aforementioned potential negative consequences of both low and high ACC values. Moreover, it would be good to first investigate the genetic correlations between ACC and other breeding goal traits before adding ACC to the breeding goal.

### Future research

In this study, we only investigated the relationship between ACC and the additive genetic variance. Future research could explore whether the additive genetic variance may also be affected by the distribution of crossovers along the chromosome. This could for example be done by incorporating measures of intrachromosomal genetic shuffling [41], which could provide insights into the potential importance of crossover positions in breeding programs, as genetic shuffling has been shown to be heritable in pigs [13]. Such studies may help to clarify whether certain combinations of the number and position of crossovers, rather than total ACC alone, could be exploited to increase additive genetic variance in breeding programs.

## Conclusions

We studied the link between ACC and the $\:{\sigma\:}_{a}^{2}$ using empirical data for different reproductive and production traits in pigs, by estimating $\:{\sigma\:}_{a,sire}^{2}$ within bins of individuals defined by the number of paternal autosomal crossovers they inherited. Results showed no statistically significant differences in $\:{\widehat{\sigma\:}}_{a,sire}^{2}$ across ACCs bins. Nevertheless, some patterns in $\:{\widehat{\sigma\:}}_{a,sire}^{2}$ were apparent, although these appeared to be trait-dependent and did not show a consistently increasing pattern. For reproduction traits, we generally observed that $\:{\widehat{\sigma\:}}_{a,sire}^{2}$ was maximised towards intermediate ACCs ranges, while for production traits, a higher ACCs resulted in higher $\:{\widehat{\sigma\:}}_{a,sire}^{2}$. In conclusion, ACC may be of interest in breeding programs as a trait to investigate further, although the present results do not provide significant evidence to support its direct use.

## Supplementary Information

Below is the link to the electronic supplementary material.

Supplementary Material 1

Supplementary Material 2

## Data Availability

The data supporting the findings of this study are available from Topigs Norsvin. However, restrictions apply, as these data were used under license for the current study and are not publicly available.
