# Supplementary material for: Potential of autosomal crossover count to enhance additive genetic variance in pig breeding programs
Source: Genet Sel Evol. 2026 Nov 10;58:85. doi: 10.1186/s12711-026-01114-0 (PMC13753163; doi:10.1186/s12711-026-01114-0)

Additional file 1

Figure *S1*. Patterns of $\hat{\sigma}^2_{a,sire}$ and $\hat{\sigma}^2_{a,dam}$ for univariate analysis across bins of autosomal crossover count

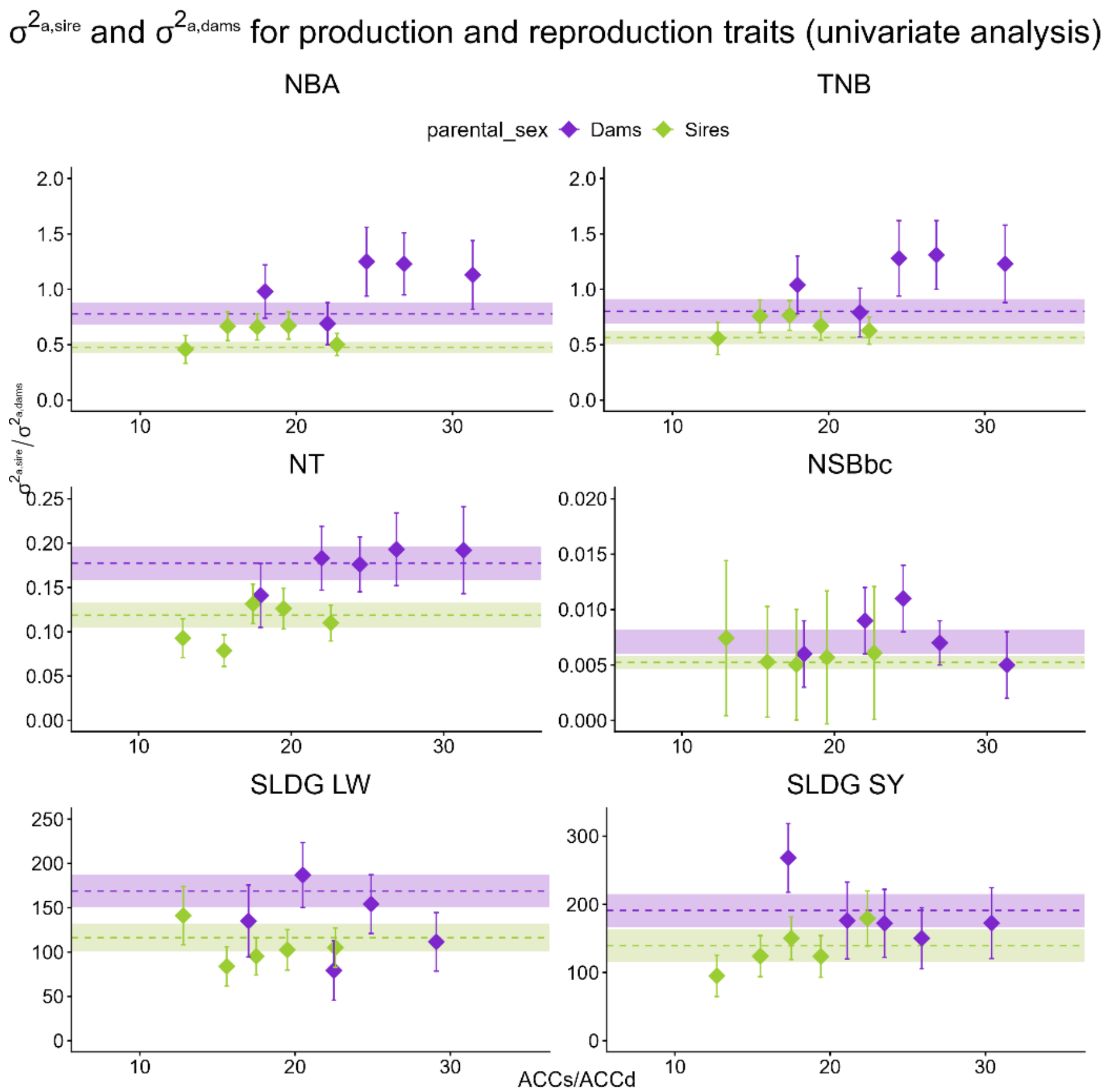

Figure *S2*. $\hat{\sigma}^2_{a,sire}$ across all the range of ACCs using random regression models in NBA and TNB

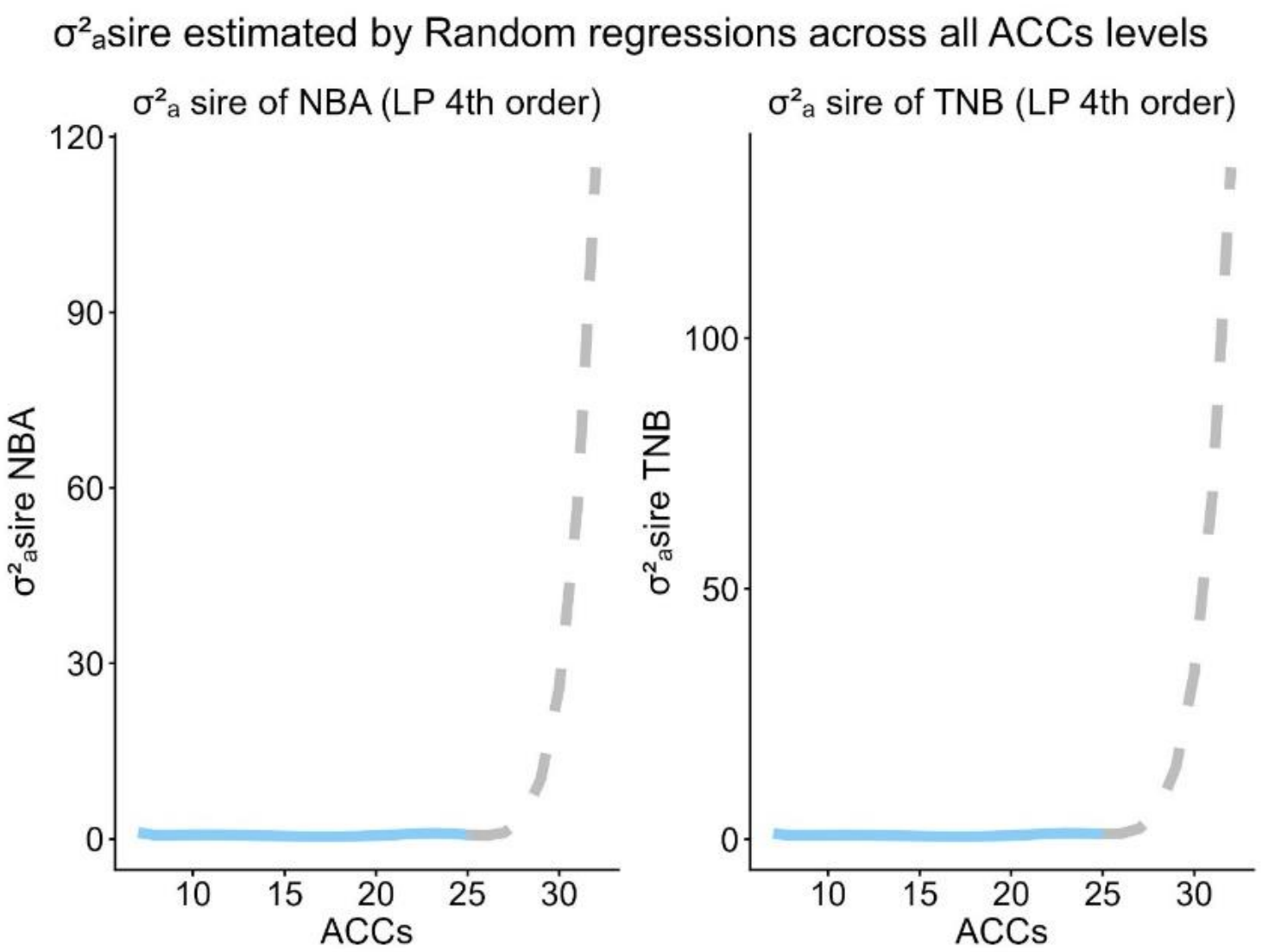

Supplement: Supplementary file 1 [file 12711_2026_1114_MOESM1_ESM.pdf]
