# Supplementary material for: Potential of autosomal crossover count to enhance additive genetic variance in pig breeding programs
Source: Genet Sel Evol. 2026 Nov 10;58:85. doi: 10.1186/s12711-026-01114-0 (PMC13753163; doi:10.1186/s12711-026-01114-0)
Supplement: Supplementary file 2 [file 12711_2026_1114_MOESM2_ESM.pdf]

Additional file 2

Table *S1*. Estimated genetic parameters and their standard errors, shown for each trait and bin from the univariate analyses of sire models.

Trait	Estimated genetic parameters from the univariate analysis using all the dataset				
	h^2 (SE)	σ_p^2 (SE)	σ_{pe}^2 (SE)	σ_e^2 (SE)	
NBA	0.14 (0.02)	13.88 (0.09)	0.94 (0.05)	11.79 (0.08)	
TNB	0.15 (0.02)	15.15 (0.10)	1.19 (0.06)	12.58 (0.08)	
NT	0.46 (0.05)	1.04 (0.05)	0.16 (0.01)	0.74 (0.01)	
NSBbc	0.110 (0.014)	0.191 (0.001)	0.011 (0.001)	0.167 (0.001)	
SLDG (sow line)	0.20 (0.03)	2281.48 (14.18)	-	1523.18 (12.83)	
SLDG (boar line)	0.16 (0.02)	3485.66 (26.29)	-	2094.37 (19.25)	
	h^2 (SE) from univariate analysis using bins				
	Bin 1	Bin 2	Bin 3	Bin 4	Bin 5
NBA	0.13 (0.03)	0.19 (0.04)	0.19 (0.04)	0.20 (0.03)	0.13 (0.03)
TNB	0.14 (0.04)	0.20 (0.04)	0.20 (0.03)	0.18 (0.03)	0.16 (0.03)
NT	0.38 (0.09)	0.31 (0.07)	0.49 (0.08)	0.49 (0.09)	0.43 (0.08)
NSBbc	0.15 (0.02)	0.11 (0.02)	0.10 (0.02)	0.13 (0.02)	0.13 (0.02)
SLDG (sow line)	0.24 (0.07)	0.15 (0.04)	0.17 (0.04)	0.18 (0.04)	0.18 (0.04)
SLDG (boar line)	0.11 (0.03)	0.14 (0.03)	0.17 (0.04)	0.14 (0.04)	0.21 (0.05)
	σ_p^2 (SE) from univariate analysis using bins				
	Bin 1	Bin 2	Bin 3	Bin 4	Bin 5
NBA	14.63 (0.24)	13.90 (0.20)	13.71 (0.18)	13.72 (0.19)	14.10 (0.18)
TNB	15.79 (0.26)	15.12 (0.22)	14.92 (0.20)	14.99 (0.20)	15.45 (0.20)
NT	0.96 (0.03)	1.02 (0.03)	1.06 (0.03)	1.04 (0.03)	1.02 (0.02)
NSBbc	0.189 (0.003)	0.190 (0.003)	0.191 (0.003)	0.191 (0.003)	0.190 (0.002)
SLDG (sow line)	2256.20 (43.08)	2284.74 (38.08)	2250.59 (33.11)	2307.92 (35.62)	2300.36 (33.51)
SLDG (boar line)	3475.85 (61.09)	3549.65 (58.05)	3480.06 (54.08)	3482.71 (59.75)	3473.64 (62.72)
	σ_{pe}^2 (SE) from univariate analysis using bins				
	Bin 1	Bin 2	Bin 3	Bin 4	Bin 5
NBA	1.98 (0.19)	1.78 (0.16)	1.86 (0.14)	1.65 (0.14)	1.79 (0.14)
TNB	2.46 (0.22)	2.25 (0.18)	2.13 (0.16)	2.05 (0.16)	2.34 (0.16)
NT	0.16 (0.04)	0.12 (0.03)	0.24 (0.03)	0.16 (0.03)	0.11 (0.02)
NSBbc	0.019 (0.002)	0.019 (0.002)	0.016 (0.002)	0.017 (0.002)	0.015 (0.002)
	σ_e^2 (SE) from univariate analysis using bins				
	Bin 1	Bin 2	Bin 3	Bin 4	Bin 5
NBA	11.61 (0.23)	11.03 (0.19)	10.84 (0.17)	11.02 (0.18)	11.35 (0.17)
TNB	12.25 (0.25)	11.59 (0.20)	11.47 (0.17)	11.84 (0.18)	12.05 (0.18)

NT	0.72 (0.04)	0.79 (0.03)	0.67 (0.03)	0.72 (0.03)	0.75 (0.03)
NSBbc	0.16 (0.003)	0.16 (0.002)	0.16 (0.002)	0.16 (0.002)	0.16 (0.002)
SLDG (sow line)	1498.0 (58.46)	1526.77 (49.79)	1519.17 (38.85)	1495.43 (42.21)	1623.89 (39.01)
SLDG (boar line)	1871.45 (69.49)	2050.71 (65.58)	2126.79 (61.20)	2115.57 (72.70)	2078.34 (78.39)

Table *S2*. Genetic correlations and their standard errors between bins from the bivariate analysis for each trait [a)].

NBA					
Bin	1	2	3	4	5
1	1	0.99 (0.08)	0.99 (0.08)	0.99 (0.07)	0.99 (0.10)
2		1	0.83 (0.08)	0.91 (0.07)	0.92 (0.08)
3			1	0.89 (0.07)	0.95 (0.05)
4				1	0.91 (0.06)
5					1
TNB					
Bin	1	2	3	4	5
1	1	0.87 (0.12)	0.99 (0.07)	0.99 (0.10)	0.98 (0.10)
2		1	0.79 (0.09)	0.99 (0.07)	0.93 (0.08)
3			1	0.96 (0.06)	0.96 (0.05)
4				1	-
5					1
NSBbc					
Bin	1	2	3	4	5
1	1	0.78 (0.13)	0.80 (0.12)	-	0.75 (0.12)
2		1	0.94 (0.10)	0.99 (0.09)	0.99 (0.07)
3			1	0.93 (0.08)	0.88 (0.08)
4				1	0.99 (0.06)
5					1

[a)] "-" indicates that the log-likelihood of the model did not converge

Table *S3.* Description of the bins created for the univariate analysis in dam models.

Trait	Bin 1		Bin 2		Bin 3		Bin 4		Bin 5	
	N	Min-Max ACCs	N	Min-Max ACCs	N	Min-Max ACCs	N	Min-Max ACCs	N	Min-Max ACCs
TNB/NBA	11,680	8-20	12,551	21-23	8,443	24-25	10,072	26-28	8,114	29-47
NSBbc	11,671	8-20	12,540	21-23	8,435	24-25	10,068	26-28	8,110	29-47
NT	3,482	8-20	3,662	21-23	2,495	24-25	2,971	26-28	2,317	29-47
SLDG (sow line)	6,503	6-19	8,980	20-22	6,747	23-24	8,941	25-27	8,582	28-47
SLDG (boar line)	8,800	8-19	6,035	20-21	6,304	22-23	7,601	24-26	6,428	27-44

Table *S4*. Estimated genetic parameters and their standard errors, shown for each trait and bin from the univariate analyses of dam models.

Trait	Estimated genetic parameters from the univariate analysis using all the dataset				
	$\sigma^2_{a,dam}$ (SE)	h^2 (SE)	σ^2_p (SE)	σ^2_{pe} (SE)	σ^2_e (SE)
NBA	0.78 (0.10)	0.22 (0.03)	13.88 (0.09)	0.56 (0.08)	11.85 (0.1)
TNB	0.80 (0.11)	0.21 (0.03)	15.11 (0.09)	0.82 (0.09)	12.65 (0.08)
NT	0.18 (0.02)	0.69 (0.07)	1.02 (0.08)	0.05 (0.01)	0.75 (0.01)
NSBbc	0.007 (0.001)	0.15 (0.02)	0.191 (0.001)	0.007 (0.001)	0.168 (0.001)
SLDG (LW)	168.86 (18.18)	0.30 (0.03)	2262.41 (16.05)		1522.25 (12.83)
SLDG (SY)	191.16 (24.02)	0.22 (0.03)	3456.90 (26.07)		2095.40 (19.26)
	$\sigma^2_{a,dam}$ (SE) from univariate models using bins				
	Bin 1	Bin 2	Bin 3	Bin 4	Bin 5
NBA	0.98 (0.24)	0.69 (0.19)	1.25 (0.31)	1.23 (0.28)	1.13 (0.31)
TNB	1.04 (0.26)	0.79 (0.22)	1.28 (0.34)	1.31 (0.31)	1.23 (0.35)
NT	0.14 (0.04)	0.18 (0.04)	0.18 (0.03)	0.19 (0.04)	0.19 (0.05)
NSBbc	0.006 (0.003)	0.009 (0.003)	0.011 (0.003)	0.007 (0.002)	0.005 (0.002)
SLDG (sow line)	135,03 (40.55)	186,91 (36.65)	79,24 (33.58)	154,17 (33.08)	111,56 (33.01)
SLDG (boar line)	268,20 (50.41)	176,16 (56.28)	172,18 (49.76)	150,26 (44.72)	172,45 (51.79)
	h^2 (SE) from univariate analysis using bins				
	Bin 1	Bin 2	Bin 3	Bin 4	Bin 5
NBA	0.28 (0.07)	0.20 (0.06)	0.36 (0.09)	0.35 (0.08)	0.32 (0.09)
TNB	0.27 (0.07)	0.21 (0.06)	0.34 (0.09)	0.34 (0.08)	0.33 (0.09)
NT	0.54 (0.14)	0.73 (0.14)	0.70 (0.12)	0.78 (0.17)	0.72 (0.18)
NSBbc	0.13 (0.05)	0.19 (0.05)	0.23 (0.07)	0.14 (0.05)	0.10 (0.06)
SLDG (sow line)	0.24 (0.07)	0.33 (0.07)	0.15 (0.06)	0.27 (0.06)	0.19 (0.06)
SLDG (boar line)	0.31 (0.06)	0.20 (0.06)	0.20 (0.06)	0.17 (0.06)	0.20 (0.06)
	σ^2_p (SE) from univariate analysis using bins				
	Bin 1	Bin 2	Bin 3	Bin 4	Bin 5
NBA	14.04 (0.18)	13.80 (0.17)	14.02 (0.22)	13.97 (0.20)	13.91 (0.22)
TNB	15.26 (0.20)	15.15 (0.19)	15.10 (0.23)	15.24 (0.21)	15.06 (0.24)
NT	1.04 (0.03)	1.00 (0.02)	1.01 (0.03)	1.00 (0.03)	1.07 (0.03)

NSBbc	0.190 (0.002)	0.191 (0.002)	0.190 (0.003)	0.190 (0.003)	0.190 (0.003)
SLDG (sow line)	2244.03 (29.35)	2252.54 (33.62)	2158.41 (37.16)	2267.15 (33.91)	2357.39 (35.99)
SLDG (boar line)	3436.55 (51.81)	3465.82 (63.09)	3416.95 (60.86)	3496.39 (56.71)	3504.28 (56.52)
	σ^2_{pe} (SE) from univariate analysis using bins				
	Bin 1	Bin 2	Bin 3	Bin 4	Bin 5
NBA	1.20 (0.23)	1.39 (0.20)	1.49 (0.29)	0.93 (0.26)	1.31 (0.29)
TNB	1.60 (0.26)	1.82 (0.23)	1.95 (0.33)	1.41 (0.28)	1.59 (0.33)
NT	0.10 (0.03)	0.01 (0.03)	0 [a)]	0.07 (0.04)	0.07 (0.05)
NSBbc	0.015 (0.003)	0.011 (0.003)	0.014 (0.003)	0.013 (0.003)	0.015 (0.003)

[a)] "0" indicates that the variance component was estimated at boundary.

Table *S5*. Log-likelihood values for each polynomial order and trait, with p-values from likelihood ratio tests between consecutive models.

Trait	Order	LogL	LRT – p-value; df*; (alpha = 0.05)
TNB	0	-92,348.62	
	1	-92,330.98	2.18e-08
	2	-92,320.74	1.35e-04
	3	-92,310.35	3.50e-04
	**4**	**-92,302.92**	**1.09e-2**
NBA	0	-90,472.54	-
	1	-90,458.81	1.09e-06
	2	-90,452.44	5.23e-03
	3	-90,444.11	2.25e-03
	**4**	**-90,433.46**	**7.10e-04**
NT	**0**	**-6,646.61**	-
	1	-6,646.01	0.54881
NSBbc	**0**	**17,966.49**	**-**
	1	17,967.86	0.254107
SLDG (LW)	**0**	**-167,753.9**	**-**
	1	-167,752.8	0.34646
SLDG (SY)	**0**	**-156,758.7**	**-**
	1	-156,758.3	0.66365

* Likelihood ratio tests compared consecutive polynomial orders, with degrees of freedom (df) equal to the number of additional sire polynomial coefficients and their covariances estimated in the more complex model.

Table *S6*. Phenotypic means and their standard errors, shown for each trait and bin from the univariate analyses of sire models.

Trait	Phenotypic mean (SE)				
	Bin 1	Bin 2	Bin 3	Bin 4	Bin 5
NBA	14.7 (0.05)	14.7 (0.04)	14.7 (0.04)	14.7 (0.04)	14.6 (0.03)
TNB	16.1 (0.05)	16.1 (0.04)	16.0 (0.04)	16.1 (0.04)	16.0 (0.04)
NT	16.2 (0.02)	16.2 (0.02)	16.2 (0.02)	16.3 (0.02)	16.2 (0.02)

NSBbc	0.481 (0.005)	0.495 (0.005)	0.485 (0.004)	0.494 (0.004)	0.478 (0.004)
SLDG (LW)	692.0 (0.83)	690.5 (0.74)	690.9 (0.65)	691.4 (0.68)	690.3 (0.65)
SLDG (SY)	822.3 (0.81)	822.1 (0.77)	822.7 (0.72)	822.1 (0.80)	821.9 (0.83)
